# Machine Learning Prediction of *Clostridioides difficile* Infection in Hospitalized COVID-19 Patients Across Pandemic Waves

**DOI:** 10.3390/healthcare14131869

**Published:** 2026-06-26

**Authors:** Oliver Lohaj, Pavel Kočan, Anna Biceková, Daniela Javorská

**Affiliations:** 1 Institute of Artificial Intelligence, Faculty of Electrical Engineering and Informatics, Technical University of Košice, Letná 9, 040 01 Košice, Slovakia; pavel.kocan@student.tuke.sk (P.K.); anna.bicekova@tuke.sk (A.B.); 2 Department of Infectiology and Travel Medicine, Faculty of Medicine, L. Pasteur University Hospital, Pavol Jozef Šafárik University in Košice, 040 01 Košice, Slovakia; daniela.javorska@student.upjs.sk

**Keywords:** *Clostridioides difficile* infection, COVID-19, machine learning, predictive models, interpretability, SHAP, XGBoost, explainability, clinical decision support

## Abstract

**Highlights:**

**What are the main findings?**

- Machine learning models predicted *Clostridioides difficile* infection risk in hospitalized COVID-19 patients across pandemic waves.
- The models achieved more than a fivefold improvement over the random PR-AUC baseline, with inflammatory markers identified as important predictors.

**What are the implications of the main findings?**

- Interpretable models supported by SHAP and LIME can provide transparent, patient-specific CDI risk explanations.
- The developed web-based decision-support tool showed promising preliminary usability for clinical application.

**Abstract:**

**Background/Objectives**: *Clostridioides difficile* infection (CDI) represents an important healthcare-associated complication in hospitalized patients, particularly in those exposed to antibiotics, prolonged hospitalization, and intensive treatment during COVID-19. This study aimed to design, evaluate, and interpret machine learning models for predicting CDI occurrence in hospitalized COVID-19 patients across individual pandemic waves, with respect to administered treatment and clinical characteristics. **Methods**: Anonymized clinical data from 3848 COVID-19-positive patients treated at the University Hospital of L. Pasteur in Košice, Slovakia, were analyzed following the CRISP-DM methodology. Four classification models were compared: logistic regression, Random Forest, XGBoost, and a multilayer perceptron. Missing values were addressed using MICE and KNN imputation, and class imbalance was handled through oversampling techniques. Given the low CDI prevalence of 2.68%, model performance was primarily assessed using the precision–recall area under the curve (PR-AUC), with AUROC reported for comparability. Interpretability was supported using SHAP, LIME, and odds ratio analysis. **Results**: The best-performing models achieved PR-AUC values up to 0.160, representing more than a fivefold improvement over the random baseline of 0.027. XGBoost reached the highest AUROC of 0.823, followed by Random Forest with 0.798. Inflammatory markers were identified as important predictors of CDI risk. A Flask-based decision-support web application was developed to provide CDI risk estimation with patient-specific explanations. A preliminary pilot usability evaluation involving two physicians yielded a mean System Usability Scale score of 73.75; however, the very small evaluator sample limits the generalizability of this finding. **Conclusions**: Interpretable machine learning models can support clinically meaningful CDI risk stratification in highly imbalanced COVID-19 hospital datasets. The proposed decision-support tool shows potential for future integration into clinical workflows, although external and prospective validation is required.

## 1. Introduction

The COVID-19 pandemic placed extraordinary pressure on healthcare systems worldwide and exposed hospitalized patients to a wide range of secondary complications. Among the clinically significant healthcare-associated conditions observed during this period was *Clostridioides difficile* infection (CDI), which is strongly associated with antibiotic therapy, prolonged hospitalization, advanced age, comorbidities, and compromised immune status [1,2,3]. Patients hospitalized with COVID-19 frequently received intensive pharmacological treatment, including broad-spectrum antibiotics, antivirals, anticoagulants, proton pump inhibitors, and immunomodulatory agents. This treatment context, together with prolonged hospital stays and infection-control challenges during pandemic waves, may have increased the risk of CDI development [4]. Beyond antibiotic exposure, severe COVID-19 may further increase susceptibility to CDI through systemic inflammation, intestinal dysbiosis, and disruption of the intestinal epithelial barrier, which can increase permeability and reduce colonization resistance against opportunistic pathogens such as *C. difficile* [5,6].

CDI is caused by an anaerobic, spore-forming bacterium and represents one of the leading causes of healthcare-associated antibiotic-associated diarrhea and colitis [3]. Its spores are highly resistant to standard disinfectants and can persist in hospital environments, facilitating transmission among vulnerable patients. Clinical manifestations range from mild diarrhea to severe colitis, toxic megacolon, septic shock, and death [3]. The clinical importance of CDI is further emphasized by epidemiological trends. National data from Slovakia indicate a marked increase in CDI cases over the last decade, while international studies have reported extensive antibiotic use among COVID-19 patients despite a substantially lower proportion of confirmed bacterial co-infections [4]. These observations highlight the need for improved risk stratification tools that can support timely identification of patients at increased risk of CDI.

Machine learning methods have increasingly been investigated for CDI prediction. A systematic review of machine learning-based CDI prediction models identified several retrospective studies, most of which were conducted in the United States and Asia [7], Ötleş et al. [8] developed a machine learning approach for hospital-onset CDI surveillance, while Panchavati et al. [9] compared several machine learning algorithms for predicting CDI risk in hospitalized patients. Commonly used algorithms included logistic regression, Random Forest, gradient boosting methods, support vector machines, and neural networks, with reported AUROC values typically ranging from approximately 0.60 to 0.81 [7]. Frequently identified predictors included age, recent hospitalization, antibiotic exposure, proton pump inhibitor use, comorbidities, immunosuppressive therapy, and previous CDI history [7]. These predictors correspond well with established clinical risk factors and demonstrate the potential of data-driven models to support CDI risk assessment.

More recent studies have reported promising results using large-scale datasets and advanced machine learning or deep learning approaches. Pham et al. developed prediction models based on demographic variables and medication history in a large longitudinal cohort and reported AUROC values above 0.85 for ensemble-based approaches [10]. Marra et al., however, reported more modest predictive performance in a routine hospital cohort, illustrating the difficulty of CDI prediction in real-world clinical datasets with limited positive cases [11]. Panchavati et al. analyzed a large multicenter hospitalization dataset and showed that XGBoost and deep learning architectures achieved AUROC values around 0.80, while SHAP analysis identified laboratory markers, antibiotic exposure, proton pump inhibitor use, and comorbidities as important predictors [9]. Kim et al. further demonstrated the potential of temporal deep learning models applied to longitudinal electronic health records, with strong external validation performance and clinically relevant predictors such as inflammatory and hematological markers [12]. In addition, Van Werkhoven et al. developed a logistic regression-based risk model for CDI in hospitalized patients receiving systemic antibiotics, achieving AUROC values above 0.80 in both derivation and validation cohorts [13].

Recent studies have further emphasized the importance of explainability and outcome-oriented modeling in CDI research. Madden et al. [14] developed explainable machine learning models for predicting CDI outcomes and showed that explainable prediction outputs can support clinically relevant questions at the time of CDI diagnosis, although their work focused on outcomes after CDI rather than on predicting CDI occurrence in COVID-19 patients. Chen et al. [15] systematically reviewed machine learning approaches for CDI and CDI-related outcomes, highlighting the growing use of ML in this field but also pointing to heterogeneity in study designs, outcomes, validation strategies, and reporting. Ruzicka et al. [16] developed machine learning models for recurrence and mortality outcomes after CDI using Japanese hospital data, demonstrating the potential of predictive analytics for post-diagnosis risk stratification. Together, these studies show that machine learning is increasingly relevant for CDI incidence and outcome prediction; however, they do not specifically address CDI prediction in hospitalized COVID-19 patients across pandemic waves, nor do they combine pandemic-wave context, treatment-related variables, explainability methods, and preliminary usability evaluation within a deployable clinical decision-support application.

Despite these advances, previous studies have mainly focused on general CDI prediction, antibiotic-associated CDI, or outcomes after CDI diagnosis, while CDI risk prediction in hospitalized COVID-19 patients across pandemic waves remains insufficiently explored [7,9,12,14]. Existing CDI prediction studies have largely focused on standard hospital populations and have rarely considered the specific clinical context of COVID-19 hospitalization. In particular, previous models generally do not account for pandemic-wave-specific variability, changes in treatment strategies, vaccination status, or temporal differences in patient populations [7,14]. Moreover, although predictive performance is often reported, fewer studies combine model development with interpretability methods and practical deployment in a clinical decision-support environment. Interpretability and explainability are especially important in healthcare, where clinicians need to understand not only the predicted risk but also the factors contributing to an individual prediction.

This article addresses these gaps by developing and evaluating machine learning models for CDI prediction in hospitalized COVID-19 patients across four pandemic waves: Wuhan, Alpha, Delta, and Omicron in Central Europe. The study uses anonymized clinical data from the University Hospital of L. Pasteur in Košice, Slovakia, and incorporates demographic, clinical, laboratory, vaccination, and treatment-related variables. In addition to predictive performance, the proposed framework emphasizes interpretability through SHAP, LIME, and odds ratio analysis. The resulting models are further integrated into a Flask-based web application designed to provide patient-specific CDI risk estimation and support clinical decision-making.

The main contributions of this study are as follows: first, it investigates CDI prediction in the specific context of hospitalized COVID-19 patients across individual pandemic waves in a Central European clinical setting; second, it evaluates a complete machine learning pipeline including missing-value imputation, feature selection, class imbalance handling, probability calibration, and threshold optimization; third, it integrates multiple explainability approaches to support transparent and patient-specific explanations; and fourth, it demonstrates practical applicability through the development and preliminary usability evaluation of a web-based clinical decision-support tool.

Therefore, the novelty of this study lies not only in applying machine learning to CDI prediction, but in addressing CDI risk in the specific context of hospitalized COVID-19 patients across pandemic waves, while integrating explainability and preliminary usability evaluation into a deployable clinical decision-support workflow.

## 2. Materials and Methods

### 2.1. Study Design and Data

This study employed a retrospective observational design following the CRISP-DM methodology [17]. The CRISP-DM framework was used to structure the analytical process from the initial understanding of the clinical problem and available data, through data preparation and modeling, to the evaluation and deployment of the resulting predictive models. This approach was selected because it provides a systematic and reproducible workflow for data analytics projects and is well suited for clinical datasets that require careful preprocessing, feature selection, and model validation.

Anonymized clinical data were obtained from University Hospital L. Pasteur in Košice, Slovakia. The dataset included records of COVID-19-positive patients hospitalized during the pandemic period and reflected real-world clinical practice in a tertiary healthcare facility. Patient records were divided into four subsets according to the dominant COVID-19 pandemic waves: Wuhan, Alpha, Delta, and Omicron. This division enabled the analysis to account for temporal differences in patient characteristics, treatment strategies, vaccination status, and clinical management across the pandemic period. The distribution of patients across individual waves is presented in Table 1.

The final dataset comprised 3848 hospitalized COVID-19-positive patients. The target variable was the diagnosis of *Clostridioides difficile* infection, identified using the ICD-10 code A04.7. For modeling purposes, the outcome was transformed into a binary classification label, distinguishing CDI-positive patients from CDI-negative patients. Patients with a recorded CDI diagnosis were assigned to the positive class, while all remaining hospitalized COVID-19 patients were assigned to the negative class. Overall, 103 patients were identified as CDI-positive, corresponding to a prevalence of 2.68% in the analyzed dataset. This low prevalence resulted in a highly imbalanced classification problem, which was explicitly considered during model development and evaluation.

### 2.2. Features

The dataset included a broad range of variables reflecting demographic characteristics, lifestyle factors, pre-existing health status, laboratory findings, COVID-19-related information, and administered treatment. Demographic variables included age and sex, while social factors included smoking status and alcohol consumption. Comorbidities were represented by binary indicators describing the presence or absence of selected chronic conditions, including hypertension, diabetes mellitus, cardiovascular diseases, respiratory diseases, renal diseases, hepatic diseases, oncological diseases, and immunosuppression.

Laboratory parameters covered several clinically relevant categories, including hematological, inflammatory, coagulation, metabolic, renal, hepatic, electrolyte, and immunological markers. Non-routine variables, including vitamin D, were retained because they were available in the retrospective dataset and may reflect immune, inflammatory, or nutritional status; however, their inclusion was exploratory rather than a requirement for routine clinical implementation. These variables were included because they may reflect systemic inflammation, organ dysfunction, immune response, and overall disease severity, all of which can be relevant in the context of CDI risk among hospitalized COVID-19 patients. In addition to laboratory and clinical variables, the dataset also included vaccination status, prior COVID-19 infection, and administered medications. Medication-related variables included antibiotics, antivirals, immunosuppressants, anticoagulants, and proton pump inhibitors, as these therapies may influence CDI risk either directly, through disruption of intestinal microbiota, or indirectly, through their association with disease severity and hospitalization course.

Because laboratory values were recorded repeatedly during hospitalization, time-series measurements were transformed into summary features to make them suitable for tabular machine learning models. For each selected laboratory parameter, four aggregate statistics were calculated: the first recorded value, the last recorded value, the minimum value, and the maximum value during the hospitalization period. This approach was selected as a pragmatic way to transform longitudinal laboratory measurements into a fixed-length tabular representation suitable for the evaluated machine learning models. The first recorded value reflects the earliest available clinical status, the last recorded value reflects the most recent available status, and the minimum and maximum values capture clinically relevant extremes that may indicate disease severity or systemic inflammatory response during hospitalization.

The CDI label refers to confirmed CDI occurrence during the hospitalization episode. In routine clinical practice, CDI testing and laboratory confirmation may require additional processing time; therefore, in some cases, the confirmed CDI diagnosis may have been entered into the hospital record retrospectively after patient transfer, discharge, or death. However, such patients were considered CDI-positive only when CDI was confirmed as occurring during the hospitalization episode. Therefore, the laboratory aggregates used in this study characterize the clinical course of the hospitalization episode rather than a strictly admission-time prediction window.

### 2.3. Data Preprocessing

All wave-specific datasets were merged into a single analytical dataset. To preserve the temporal context of the pandemic, an additional categorical feature representing the COVID-19 pandemic wave was created and included in the modeling dataset. This enabled the models to account for potential differences between waves, such as changes in patient characteristics, dominant viral variants, vaccination coverage, and treatment strategies.

Before modeling, the dataset underwent several preprocessing steps to improve data quality and reduce the risk of biased model estimates. Attributes with more than 20% missing values were excluded, as a high proportion of missingness could reduce the reliability of imputation and weaken the interpretability of the resulting models. Physiologically implausible values were also identified and corrected where possible. For example, oxygen saturation values above 100% were considered invalid and were either corrected or replaced with missing values before imputation. This step was necessary to minimize the influence of data entry errors and measurement inconsistencies commonly present in retrospective clinical datasets.

The dataset was then divided into training and test subsets using stratified sampling to preserve the proportion of CDI-positive and CDI-negative cases in both subsets. First, 80% of the data were assigned to the training set and 20% to the independent test set. The training set was subsequently divided into a reduced training set and a calibration set using an 80:20 split. This resulted in an effective division of 64% of the data for model training, 16% for probability calibration, and 20% for final model testing. The independent test set was kept separate throughout the model development process and was used only for final performance evaluation.

Missing values were handled using two complementary imputation strategies. The first approach was Multiple Imputation by Chained Equations (MICE), implemented using scikit-learn’s IterativeImputer (version 1.4.1.post1) with a maximum of 20 iterations [18]. This method estimates missing values by modeling each variable with missing data as a function of the remaining variables. The second approach was k-Nearest Neighbor (k-NN) imputation with k = 3 and uniform weights, where missing values are estimated based on the most similar observations in the dataset. The use of both approaches enabled comparison of whether the selected imputation strategy affected feature selection and predictive performance.

To prevent data leakage, all preprocessing steps requiring parameter estimation were fitted exclusively on the training data. The learned imputation parameters were then applied to the calibration and test sets. This procedure ensured that information from the calibration or test sets did not influence the training process and that the reported performance reflected the model’s ability to generalize to unseen patient records.

### 2.4. Feature Selection

Laboratory feature selection was performed using two complementary statistical approaches applied exclusively to the training data. This restriction was used to avoid information leakage from the calibration and test sets into the feature selection process. The aim of feature selection was to reduce dimensionality, improve model interpretability, and retain laboratory parameters with the strongest statistical association with CDI occurrence.

First, the Mann–Whitney U test was used to assess distributional differences in laboratory values between CDI-positive and CDI-negative patients. This non-parametric test was selected because many clinical laboratory variables do not follow a normal distribution and may contain skewed values or outliers. To control for multiple testing, Benjamini–Hochberg false discovery rate (FDR) correction was applied, with the significance threshold set at α = 0.05. Second, point-biserial correlation was used to quantify the strength and direction of association between continuous laboratory variables and the binary CDI target. A minimum effect size threshold of |r| ≥ 0.05 was applied to exclude variables with negligible associations, even if statistically significant.

A composite ranking score was then defined to combine statistical significance and effect size into a single feature selection criterion. The score was calculated as the sum of ${-\log}_{10}\left(q\text{-}value\right)$ values derived from the statistical tests, with the correlation component additionally weighted by the absolute value of the point-biserial correlation coefficient. This approach prioritized laboratory variables that were both statistically significant and meaningfully associated with CDI status. Based on this ranking, the top 15 laboratory features were selected for subsequent model development. This reduced the number of input variables while preserving the most informative predictors and improving the transparency and reproducibility of the modeling pipeline.

Medication-related features were reduced through exploratory analysis by comparing their frequency between CDI-positive and CDI-negative patients. Variables with observable differences between the two groups were retained, particularly when they were clinically relevant to CDI risk, such as antibiotic exposure, proton pump inhibitor use, immunosuppressive treatment, and other therapies associated with hospitalization severity or microbiome disruption.

As an additional exploratory analysis, crude odds ratios with 95% confidence intervals were calculated to compare CDI occurrence across pandemic waves, using the Wuhan wave as the reference category. These analyses were unadjusted and were intended to provide descriptive context rather than estimate an independent causal effect of pandemic wave (Supplementary Table S2).

In addition to selecting individual predictors, a composite risk score (Skóre_final) was constructed to provide an interpretable summary of patient risk. Selected laboratory variables were transformed into binary indicators using thresholds derived from ROC analysis based on the Youden index. Binary clinical variables, including comorbidities and medications, were included when they showed a significant association with CDI after FDR correction and an odds ratio greater than 1.2. The final score was calculated as the sum of all selected risk indicators. This approach enabled the transformation of heterogeneous clinical, laboratory, and treatment-related variables into a single interpretable risk score that could be used in subsequent modeling and integrated into the decision-support application.

### 2.5. Classification Models

Four classification algorithms were implemented and evaluated to compare linear, ensemble-based, and neural network approaches for CDI prediction. Logistic Regression was used as a baseline model because of its interpretability, computational efficiency, and frequent use in clinical prediction modeling. Inputs were standardized before training to ensure that variables measured on different scales contributed appropriately to the model. Random Forest was included as a tree-based ensemble method capable of modeling nonlinear relationships and interactions between predictors through bootstrap aggregation and random feature selection. XGBoost was evaluated as a gradient-boosted decision tree method with built-in regularization, which is often effective for structured tabular clinical data. Finally, a multilayer perceptron (MLP) neural network was implemented to assess whether a nonlinear neural architecture could capture more complex patterns in the dataset when trained on standardized inputs.

A major methodological challenge was the severe class imbalance in the dataset, with an approximate 97:3 ratio between CDI-negative and CDI-positive patients. To address this imbalance, several oversampling techniques were tested, including RandomOverSampler (ROS), Synthetic Minority Oversampling Technique (SMOTE), and Adaptive Synthetic Sampling (ADASYN). These methods were applied within the cross-validation pipeline to prevent data leakage and to ensure that synthetic or duplicated minority class samples were generated only from the training folds. This design allowed the models to learn from a more balanced representation of CDI-positive cases while preserving the independence of validation folds during hyperparameter tuning. In addition to ROS, SMOTE, and ADASYN, the no-oversampling setting was also evaluated; therefore, “None” in Table 2 indicates that the final reported configuration for that model performed best without oversampling, not that class imbalance was disregarded.

Hyperparameter optimization was performed using RandomizedSearchCV (version 0.14) with 5-fold stratified cross-validation. For each model, 30 randomly selected hyperparameter combinations were evaluated. Stratification was used to maintain the proportion of CDI-positive and CDI-negative cases across folds, which was particularly important due to the low number of positive cases. The optimization objective was PR-AUC, selected as the primary metric because it is more informative than accuracy or AUROC in highly imbalanced classification problems where the positive class is clinically relevant. The hyperparameter search spaces used for the evaluated models are provided in Supplementary Table S1.

After model training and hyperparameter optimization, predicted probabilities were calibrated using sigmoid-based CalibratedClassifierCV on the separate calibration set. Probability calibration was performed to improve the reliability of predicted CDI risk estimates, which is important for clinical decision-support applications where the numerical probability may influence interpretation and decision-making. Decision thresholds were then selected with the aim of prioritizing clinical sensitivity. Specifically, thresholds were chosen to achieve recall of at least 0.60 while maintaining acceptable precision. This approach reflected the clinical preference to reduce missed CDI-positive cases, while still limiting the number of false-positive alerts that could reduce the practical usefulness of the system.

### 2.6. Evaluation Metrics

Model performance was evaluated on the held-out test set using multiple complementary metrics. The test set was not used during model training, feature selection, hyperparameter optimization, or probability calibration, ensuring an unbiased assessment of final model performance on unseen patient records.

Given the severe class imbalance in the dataset, precision–recall area under the curve (PR-AUC) was used as the primary evaluation metric. PR-AUC is particularly appropriate in this context because it focuses on the performance of the model in identifying the minority positive class, which in this study corresponds to CDI-positive patients. In highly imbalanced datasets, conventional accuracy can be misleading, as a model may achieve high accuracy by predominantly predicting the majority class. Similarly, AUROC may appear favorable even when the model has limited practical ability to detect rare positive cases. Therefore, PR-AUC was prioritized as the main indicator of clinically relevant predictive performance.

AUROC was also reported to enable comparison with existing CDI prediction studies, as it remains one of the most commonly used metrics in clinical prediction modeling. While AUROC summarizes the model’s ability to discriminate between CDI-positive and CDI-negative patients across all possible thresholds, PR-AUC provides additional insight into the balance between precision and recall in the minority class. Reporting both metrics therefore provides a more comprehensive assessment of model discrimination.

Additional threshold-dependent metrics included precision, recall, and F1 score. Precision was used to quantify the proportion of predicted CDI-positive cases that were correctly classified, while recall measured the proportion of true CDI-positive cases successfully identified by the model. The F1 score was calculated as the harmonic mean of precision and recall, providing a single summary measure of their balance. Because CDI is a clinically important complication, recall was considered particularly relevant, as missed positive cases may delay appropriate monitoring or intervention.

Calibration performance was assessed using the Brier score, which measures the mean squared difference between predicted probabilities and observed outcomes. This metric was included because the developed application presents risk probabilities to users, making the reliability of probabilistic predictions important for clinical interpretation. Finally, the reference PR-AUC baseline for a random classifier was defined as the prevalence of the positive class in the test data, approximately 0.027. Model PR-AUC values were therefore interpreted relative to this baseline to determine whether the classifiers provided meaningful improvement beyond random prediction.

To quantify uncertainty in model discrimination, approximate 95% confidence intervals were calculated for AUROC values based on the composition of the held-out test set. Confidence intervals were estimated using bootstrap resampling of the held-out test set, preserving the original test set predictions and repeatedly resampling patient-level observations.

### 2.7. Explainability and Interpretability Methods

Model interpretability and explainability were addressed using three complementary approaches: SHAP, LIME, and odds ratio analysis. The aim was not only to evaluate predictive performance, but also to make the model outputs understandable and clinically meaningful for potential end users. This is particularly important in healthcare decision support, where clinicians must be able to assess why a prediction was generated before considering it in clinical reasoning.

SHAP was used to provide additive feature-attribution explanations based on the contribution of individual input variables to a specific prediction [19]. For each patient, SHAP values indicate whether selected features increased or decreased the predicted CDI risk and quantify their relative contribution to the final model output. This enabled the identification of patient-specific risk factors, such as inflammatory markers, laboratory abnormalities, comorbidities, or treatment-related variables that contributed most strongly to the predicted probability. In the application, SHAP explanations were presented to support transparent interpretation of model behavior at the individual patient level.

LIME was used as a second local explanation method to provide an independent, case-specific approximation of model behavior [20]. LIME explains individual predictions by constructing a locally interpretable surrogate model around a selected patient instance. This approach makes it possible to identify which variables were most influential for a specific prediction, even when the underlying predictive model is complex. By using both SHAP and LIME, the application provides complementary local explanations, increasing the transparency of individual CDI risk estimates and allowing users to compare whether the most influential predictors remain clinically plausible across explanation methods.

In addition to local explanation methods, odds ratio analysis was used to assess global associations between selected predictors and CDI risk. Odds ratios were derived from logistic regression coefficients and provided an interpretable measure of how individual variables were associated with increased or decreased CDI occurrence across the dataset. This global perspective complemented the patient-specific explanations generated by SHAP and LIME by identifying broader risk patterns in the analyzed population.

Together, these methods supported both local and global interpretability. SHAP and LIME enabled patient-specific explanations of individual predictions, while odds ratio analysis provided a more general view of feature associations with CDI risk. Integrating these methods into the deployed web application allowed the system to present not only a predicted CDI risk probability, but also the main factors contributing to that prediction. This explainability-oriented design was intended to improve clinical transparency, support user trust, and facilitate critical assessment of model outputs in a hospital decision-support context.

To examine the contribution of temporal and vaccination-related variables, an additional exploratory XGBoost model was trained using the selected clinical predictors together with one-hot encoded pandemic-wave indicators and vaccination status. Global SHAP values were calculated on the held-out test set. Mean absolute SHAP values were used to rank overall feature importance, while a SHAP summary plot was used to visualize the magnitude and direction of feature contributions across patients. More information is provided in Section 3.4.3.

### 2.8. Web Application

A Flask-based web application was developed to deploy the best-performing models and demonstrate their practical applicability in a clinical decision-support setting. The application enables clinicians to enter patient data through a structured interface and obtain an estimated CDI risk probability together with model-based explanations generated using SHAP and LIME. These explanations show which patient-specific variables contributed most strongly to the prediction, supporting more transparent interpretation of the model output.

In addition to the prediction module, the application includes an analytical module that provides descriptive visualizations of the dataset, allowing users to explore selected patient characteristics, laboratory parameters, and outcome distributions. The application was designed and tested for local deployment in a hospital environment, with emphasis on usability, response speed, and the ability to present prediction results in a clinically understandable form.

## 3. Results

### 3.1. Descriptive Data Analysis

**Figure 1:**
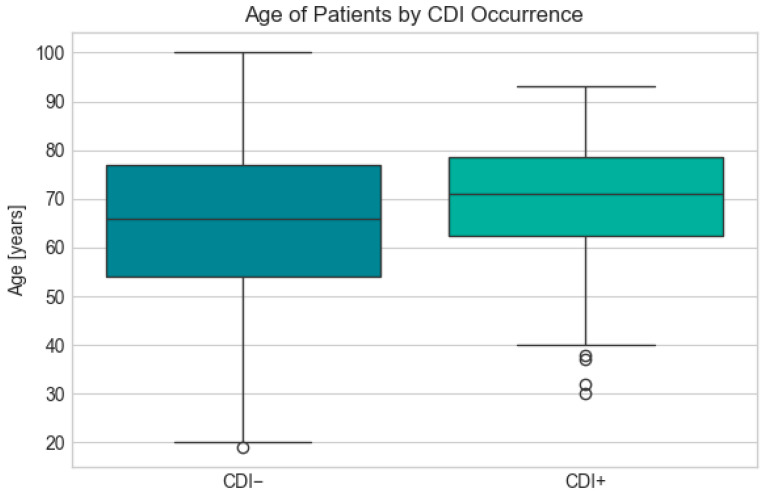
Age distribution of CDI-positive and CDI-negative patients.

CDI-positive patients were on average older than CDI-negative patients (mean age 69.3 vs. 64.8 years; median 71 vs. 66), as illustrated in Figure 1. Female patients exhibited a slightly higher CDI incidence (2.97%) compared to males (2.39%). Overall mortality was higher among CDI-positive patients (19.42%) than CDI-negative patients (11.13%). Mortality rates among CDI-positive patients varied across pandemic waves, with the highest observed during the Delta wave (33.33%), followed by Alpha (27.59%), Wuhan (15.38%), and Omicron (9.09%).

Comorbidity analysis revealed that CDI-positive patients had substantially higher prevalence of renal disease (57.28% vs. 39.15%) and cardiovascular disease (72.82% vs. 61.79%). Medication analysis indicated increased administration of antibiotics, proton pump inhibitors, and immunosuppressants in the CDI-positive group.

**Figure 2:**
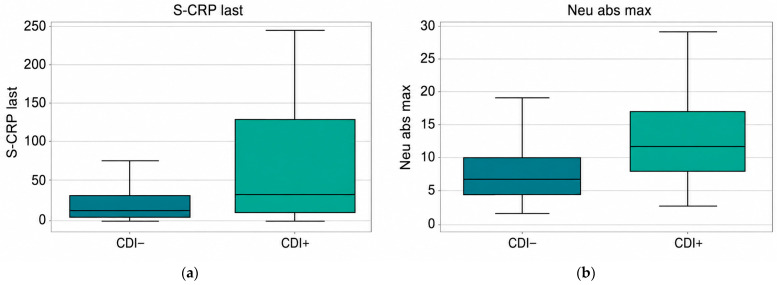
Distribution of selected inflammatory markers (NEU (**b**) and CRP (**a**)) in CDI-positive and CDI-negative patients.

Figure 2 illustrates the distribution of selected inflammatory markers between CDI-positive and CDI-negative patients. CDI-positive patients exhibited consistently elevated values of CRP and absolute neutrophil count (Neu abs), highlighting their role as key indicators of systemic inflammatory response associated with CDI.

An exploratory comparison of CDI occurrence across pandemic waves is presented in Supplementary Table S2. CDI prevalence was 2.31% during the Wuhan wave, 3.52% during Alpha, 2.31% during Delta, and 2.64% during Omicron. For example, while using Wuhan as the reference, the crude odds ratio for Omicron was 1.14 (95% CI: 0.68–1.93), indicating no clear reduction in CDI occurrence during the Omicron wave.

### 3.2. Feature Selection Results

The top 15 laboratory features selected were consistent across both imputation methods, indicating stability of the feature selection process. Inflammatory markers represented the most influential variables, with S-CRP last and Neu abs max achieving the highest composite scores (~9.0), followed by S-IL6 last (~7.6), NE/LY NLR max (~7.3), and WBC max (~7.2).

Additional relevant features included K min, D-dimer maximum, and liver enzyme values (AST min), reflecting metabolic, coagulation, and organ function alterations associated with disease severity.

The near-identical feature rankings across KNN and MICE imputation further confirm the robustness of the selection methodology and suggest that the identified predictors are driven by underlying clinical signal rather than imputation strategy.

### 3.3. Model Performance

Table 2 summarizes the performance of the best configuration for each algorithm on the test set. Confidence intervals are reported for AUROC to indicate the uncertainty of model performance estimates on the held-out test set. Oversampling was treated as a model-selection component rather than a mandatory preprocessing step. Therefore, each algorithm was evaluated with and without oversampling, and the configuration reported in Table 2 represents the best-performing setup for that model according to cross-validation and test-set performance. Consequently, “None” in Table 2 indicates that no oversampling was selected for the final configuration of that model, not that class imbalance was disregarded.

**Figure 3:**
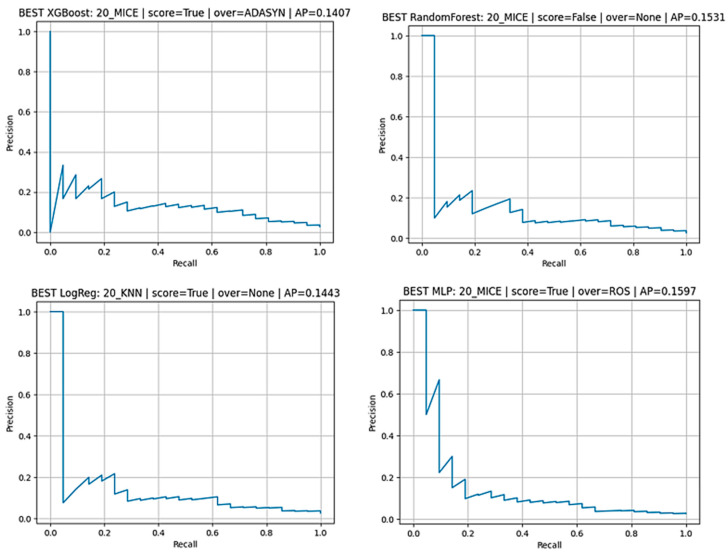
Precision–recall curves for all evaluated models on the test set.

Given the severe class imbalance, model performance was primarily evaluated using PR-AUC. All models substantially exceeded the baseline PR-AUC of 0.027, achieving values between 0.141 and 0.160, corresponding to more than a fivefold improvement over a random classifier. Precision–recall curves for all models are shown in Figure 3.

**Figure 4:**
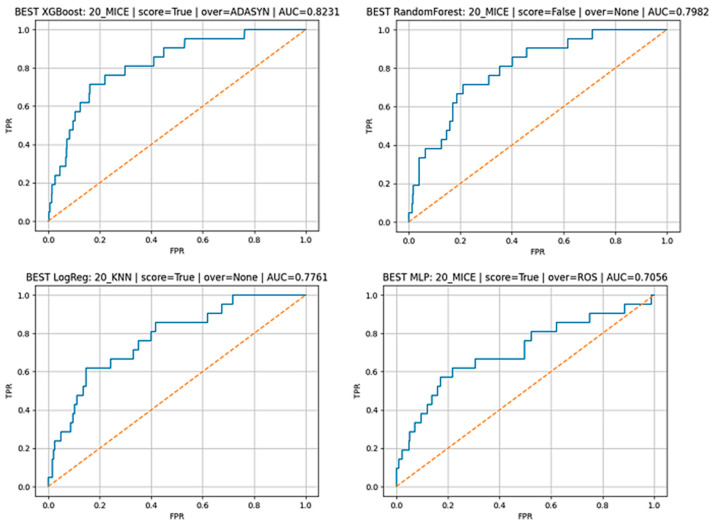
Receiver operating characteristic (ROC) curves comparing the discriminative performance of all evaluated models on the test set. The blue line shows the ROC curve of the model, a trade-off between the True Positive Rate (TPR) and False Positive Rate (FPR), while orange dashed line is the performance of random classifier.

XGBoost achieved the highest AUROC (0.823), indicating strong discriminative ability, as shown in Figure 4. The MLP achieved the highest PR-AUC (0.160); however, this was accompanied by the lowest AUROC (0.706), suggesting sensitivity to oversampling and reduced stability in probability estimation. All evaluated models demonstrated similar calibration performance, with Brier score values ranging from 0.0244 to 0.0263. The lowest Brier score was achieved by Random Forest (0.0244), indicating slightly better calibration of predicted probabilities.

### 3.4. Interpretation of Key Findings

Additional analyses were performed to assess the robustness of the proposed modeling pipeline and to further interpret the clinical relevance of the identified predictors.

#### 3.4.1. Effect of Imputation Strategy

These findings suggest that the choice of imputation method had only a limited impact on model performance. Models trained on datasets imputed using KNN and MICE showed similar rankings of the most important laboratory features and comparable performance metrics across the evaluated algorithms. The stability of feature rankings indicates that the identified predictors were not strongly dependent on the selected imputation strategy. For example, in the Random Forest model, the 20_MICE configuration achieved an AUROC of 0.798, whereas the 20_KNN configuration achieved an AUROC of 0.767, corresponding to a difference of 0.031. Similar trends were observed across other model configurations, where performance differences between the two imputation methods remained relatively small. These findings suggest that, after excluding attributes with a high proportion of missing values, both KNN and MICE imputation provided comparably suitable input data for model development. From a practical perspective, this also indicates that the predictive pipeline is reasonably robust to the choice of imputation method.

#### 3.4.2. Importance of Inflammatory Markers

The results consistently highlighted inflammatory markers among the most relevant predictors of CDI risk. Statistical association analysis performed during data preparation showed that inflammatory markers dominated the 15 most important laboratory predictors. The most relevant variables included S-CRP last, S-IL6 last, Neu abs max, NE/LY ratio (NLR) max, and WBC max. These features reflect systemic inflammatory activity and immune response, which are clinically relevant in the context of CDI and severe infectious complications. The dominance of inflammatory markers was observed consistently across the feature selection process and was further supported by local explanation methods used in the application. This finding is consistent with the known pathophysiology of CDI, which is associated with inflammatory response, intestinal damage, and systemic complications. At the same time, the presence of treatment-related and clinical variables among relevant predictors suggests that CDI risk is influenced by a combination of patient condition, immune status, and therapeutic exposure rather than by a single isolated factor.

Overall, the additional analyses confirmed the robustness of the proposed modeling pipeline and highlighted the clinical relevance of inflammatory markers as key predictors of CDI risk in hospitalized COVID-19 patients.

#### 3.4.3. Global SHAP Analysis

**Figure 5:**
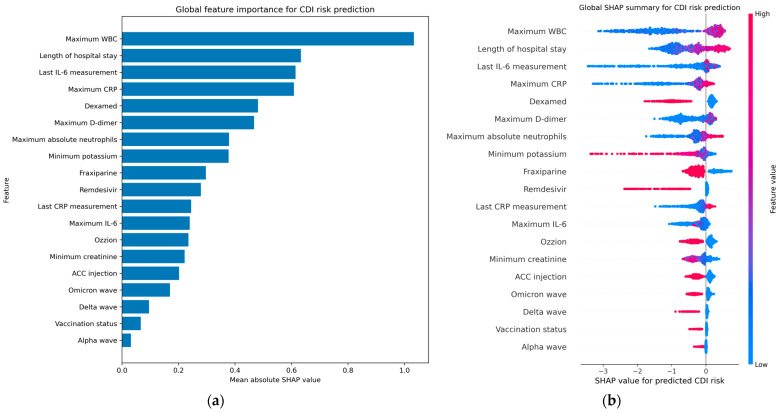
Global SHAP analysis of the exploratory XGBoost model including pandemic-wave indicators and vaccination status: (**a**) Mean absolute SHAP values showing global feature importance. Wuhan was used as the reference pandemic wave. (**b**) SHAP summary plot showing the magnitude and direction of feature contributions to predicted CDI risk.

The global SHAP analysis is presented in Figure 5. Laboratory and clinical variables contributed most strongly to the exploratory model, with maximum WBC, length of hospital stay, IL-6, CRP, D-dimer, and absolute neutrophil count among the most influential predictors. The Omicron-wave indicator was the most important temporal variable but remained less influential than the principal inflammatory and clinical predictors. Its SHAP distribution was predominantly negative, indicating that the Omicron wave was generally associated with a lower model-predicted CDI risk relative to the Wuhan reference wave. The Delta- and Alpha-wave indicators also showed comparatively low importance and predominantly negative contributions. Vaccination status had limited global importance, although vaccinated status was generally associated with a small reduction in predicted CDI risk. These findings represent model-based associations and should not be interpreted as independent causal effects.

### 3.5. Web Application Usability

**Figure 6:**
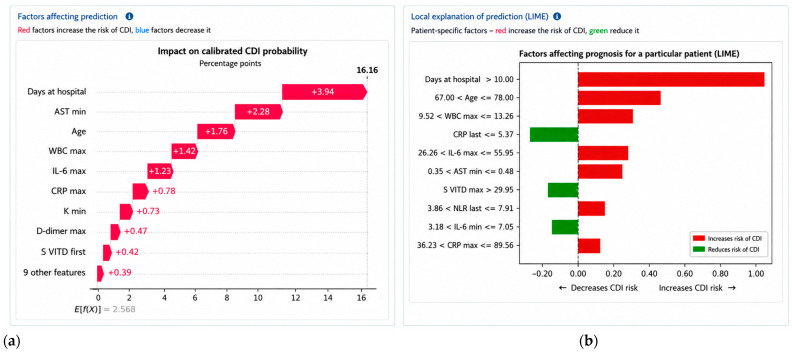
Example of an individual prediction explained using (**a**) SHAP (For this individual patient, all displayed features had positive SHAP values and therefore increased the predicted CDI risk; no risk-reducing features were present among the shown predictors) and (**b**) LIME.

The developed Flask-based web application was evaluated by two physicians from University Hospital L. Pasteur in Košice. The evaluation followed a structured testing scenario designed to simulate practical clinical use of the system. Physicians were instructed to enter anonymized patient data, execute CDI risk prediction, interpret the generated prediction outputs, and review the accompanying explainability visualizations provided by SHAP and LIME, as seen in Figure 6. In addition, participants evaluated the clarity of the user interface, overall workflow intuitiveness, response speed, and interpretability of the generated results.

Usability was assessed using the System Usability Scale (SUS), a standardized ten-item questionnaire widely used for evaluating the usability of digital systems in healthcare environments. The participating physicians assigned SUS scores of 70 and 77.5, resulting in a mean score of 73.75, which corresponds to a good level of usability and indicates suitability for further development and refinement of the application.

To complement the usability assessment, the application was also evaluated on a small set of real clinical cases. During this testing, the model correctly classified all 8 CDI-negative patients and 6 of 8 CDI-positive patients, corresponding to an overall accuracy of 87.5%. Physicians highlighted the rapid generation of predictions, the clarity of the presented results, and the availability of explainability visualizations as valuable features of the system. The explainability outputs were considered helpful for interpreting model predictions and identifying the patient-specific variables that contributed most strongly to the estimated CDI risk. Several limitations were also identified during the evaluation. Physicians noted that the number of required input variables may reduce practical usability in routine clinical workflows, especially in time-constrained hospital settings. In addition, some variables included in the predictive model, such as vitamin D levels, are not routinely measured in all hospitalized patients, potentially limiting immediate applicability in standard clinical practice. Participants also suggested that future versions of the application should include partial automation of data entry directly from electronic health records to reduce manual workload and improve workflow integration.

Given the limited number of evaluators and tested cases, these findings should be interpreted as a preliminary pilot evaluation rather than a comprehensive clinical validation. Nevertheless, the results indicate promising potential for integrating interpretable machine learning-based decision support into hospital environments and provide a foundation for future larger-scale usability and prospective clinical studies.

## 4. Discussion

This study presents a machine learning framework for CDI risk prediction in hospitalized COVID-19 patients across pandemic waves, addressing a clinically relevant and previously underexplored scenario. The results demonstrate that ensemble tree-based models, particularly XGBoost and Random Forest, provide robust predictive performance under severe class imbalance. While AUROC values (0.798–0.823) are comparable to those reported in several previous CDI prediction studies [7,9,10,11], the primary evaluation using PR-AUC offers a more informative assessment in this setting, confirming that the models achieve meaningful improvements over the random baseline. The added 95% confidence intervals for AUROC provide an estimate of uncertainty around model discrimination and support a more cautious interpretation of differences between the evaluated algorithms, particularly given the limited number of CDI-positive cases.

Compared with previous CDI-focused machine learning studies, the present work contributes a distinct clinical and methodological perspective. Madden et al. focused on explainable prediction of CDI outcomes after diagnosis, while Ruzicka et al. investigated recurrence and mortality after CDI. In contrast, our study aimed to predict CDI occurrence among hospitalized COVID-19 patients before or during clinical risk assessment, which represents a different decision-support scenario. Kim et al. developed a high-performing deep learning model for antibiotic-associated CDI using longitudinal electronic health records from Korean hospitals and achieved strong internal and external validation performance. Our study differs by focusing on a Central European COVID-19 cohort, incorporating pandemic-wave context and vaccination status, and emphasizing interpretable tabular models that were integrated into a lightweight Flask-based application. The systematic reviews by Tariq et al. and Chen et al. also highlight important limitations in the field, including heterogeneity of CDI definitions, limited real-world validation, and challenges in clinical implementation. The present study responds to these gaps by combining model development with SHAP, LIME, odds ratio analysis, calibration assessment, and preliminary usability testing with physicians. Although external multicenter validation remains necessary, the integration of explainability and usability evaluation strengthens the practical orientation of the proposed approach.

The importance of inflammatory markers (CRP, NEU, IL-6, NLR, WBC) was primarily supported by statistical association analysis, while local SHAP and LIME explanations were consistent with these findings. This is clinically consistent with the pathophysiology of CDI [3]. Elevated CRP, IL-6, neutrophil counts, NLR, and WBC likely reflect systemic inflammation, immune dysregulation, and increased disease severity. These processes may contribute to disruption of intestinal homeostasis, increased susceptibility to opportunistic pathogens, and higher risk of CDI development in hospitalized COVID-19 patients. This finding suggests that CDI risk in COVID-19 patients may be partially driven not only by antibiotic exposure but also by underlying immune dysregulation. The prominence of inflammatory markers in both statistical feature selection and local explainability analyses further supports their relevance as clinically meaningful indicators of CDI risk. Importantly, these laboratory parameters are routinely collected in clinical practice, supporting the practical applicability of the proposed model.

The exploratory wave-based analysis did not indicate a clear reduction in CDI occurrence during the Omicron wave. However, these crude estimates do not account for differences in vaccination uptake, patient characteristics, treatment practices, antibiotic exposure, or disease severity across waves. Therefore, neither pandemic wave nor vaccination status can be interpreted as independently protective based on the present analysis. A dedicated adjusted analysis would be required to distinguish the independent effects of vaccination and pandemic wave from related clinical and temporal factors.

The exploratory global SHAP analysis indicated that pandemic wave and vaccination status contributed less to CDI risk predictions than inflammatory, clinical, and treatment-related variables. Although Omicron wave and vaccinated status were generally associated with lower predicted risk, these findings reflect model-based associations and may be influenced by differences in treatment practices, patient characteristics, and vaccination uptake across waves.

The minimal impact of imputation strategy (KNN vs. MICE) on model performance highlights the robustness of the predictive pipeline. This suggests that, in this context, model performance is primarily driven by feature informativeness rather than the specific choice of imputation method, which has practical implications for deployment in heterogeneous clinical environments.

Furthermore, all evaluated models demonstrated similar calibration performance, with Brier score values ranging from 0.0244 to 0.0263. Random Forest achieved the lowest Brier score (0.0244), suggesting slightly more reliable probability estimates. This aspect is particularly important because the developed application provides individualized CDI risk probabilities rather than only binary classifications.

The inclusion of wave-specific data introduces an important temporal dimension absent from most prior CDI prediction studies [7]. Observed differences in mortality across pandemic waves (e.g., Delta 33.33% vs. Omicron 9.09% among CDI-positive patients) reflect evolving treatment strategies and patient characteristics. These findings indicate that temporal context may influence CDI risk and should be further explored in future research, for example through wave-stratified or time-aware models.

At the same time, the relatively low precision values must be considered when interpreting the practical applicability of the models. Although the models achieved substantially higher PR-AUC than the random baseline, precision remained below 10%, indicating that a considerable proportion of patients flagged as high risk would be false positives. In clinical workflows, this could contribute to unnecessary alerts, increased workload, and potential alert fatigue if the model were implemented as an automatic warning system. Therefore, the proposed model should not be interpreted as a standalone diagnostic tool or as an automatic trigger for CDI treatment. Instead, it should be considered a supportive risk-stratification tool that may help identify patients requiring closer monitoring, diagnostic consideration, or infection-control awareness, ideally in combination with clinical judgment and local workflow rules.

Several limitations must be acknowledged. First, the dataset contains a relatively small number of CDI-positive cases (*n* = 103), resulting in severe class imbalance that limits achievable precision. Second, the study is based on data from a single institution, which may restrict generalizability to other healthcare settings. Third, the inclusion of non-routine variables such as vitamin D may limit immediate clinical usability; future model refinement should therefore prioritize routinely available variables while evaluating whether predictive performance can be maintained. Another important limitation concerns the temporal structure of the laboratory data. Laboratory measurements were summarized using first, last, minimum, and maximum values during hospitalization. Although this representation captures clinically relevant changes and extremes, some values may have been recorded after CDI onset or after diagnostic sampling, because exact timing of CDI onset or diagnostic confirmation relative to every laboratory measurement was not consistently available in the retrospective dataset. Therefore, the current models should be interpreted as hospitalization-based CDI risk stratification models rather than strictly prospective admission-time prediction models. Future work should evaluate admission-only models, pre-diagnostic time-window models, or dynamic time-aware approaches that use only information available before the intended prediction time point.

The usability evaluation of the developed web application yielded a mean SUS score of 73.75, corresponding to a good usability rating. However, the small number of participants (*n* = 2) limits the generalizability of these findings, and further validation with a larger cohort of clinicians is required. Future implementation should consider integration with electronic health record systems to reduce manual data entry and improve compatibility with existing hospital workflows. The Flask application could communicate with the hospital information system through a secure application programming interface using standardized healthcare data formats such as HL7 FHIR. Routinely available demographic, laboratory, diagnostic, and medication data could then be automatically retrieved and mapped to the corresponding model inputs. Such integration would also facilitate prospective evaluation of the decision-support tool in routine clinical practice and enable its assessment in real-world decision-making scenarios.

Overall, the results demonstrate that interpretable machine learning models can provide clinically meaningful risk stratification for CDI in COVID-19 patients, even in the presence of severe class imbalance. Future work should focus on external multicenter validation using independent cohorts from hospitals with different patient populations, treatment practices, and data collection procedures. Broader usability evaluation should involve physicians from different departments, infection-control specialists, nurses, and hospital IT personnel. From an implementation perspective, integration with electronic health record systems could enable automated retrieval of routinely available demographic, laboratory, and treatment-related variables, reduce manual data entry, and improve compatibility with existing hospital workflows. Future implementation should also include threshold optimization based on clinical priorities and prospective evaluation of alert burden, false-positive rates, and user acceptance in routine hospital workflows. These steps would support prospective evaluation of the system in real-world clinical practice.

## 5. Conclusions

This study demonstrates the feasibility and clinical relevance of machine learning-based prediction of *Clostridioides difficile* infection in hospitalized COVID-19 patients. By leveraging a dataset that incorporates pandemic wave context, vaccination status, laboratory findings, comorbidities, and treatment-related variables, the study addresses a specific clinical scenario that has received limited attention in previous CDI prediction research. The inclusion of patients from a Central European healthcare setting further strengthens the originality of the work, as most existing CDI prediction studies have been conducted in different geographical, institutional, and healthcare system contexts.

The proposed models achieved meaningful predictive performance despite the severe class imbalance and low prevalence of CDI in the analyzed dataset. PR-AUC values exceeded the random baseline by more than fivefold, while AUROC values reached up to 0.823 for XGBoost. Ensemble tree-based models, particularly XGBoost and Random Forest, provided the most consistent performance and demonstrated their suitability for structured clinical data. Although the absolute PR-AUC values remained modest, the results indicate that the models were able to identify clinically relevant predictive patterns in a challenging real-world hospital dataset with a limited number of CDI-positive cases.

Statistical association analysis identified inflammatory markers, including C-reactive protein, neutrophil count, interleukin-6, neutrophil-to-lymphocyte ratio, and white blood cell count, as important predictors of CDI risk. These findings suggest that CDI development in hospitalized COVID-19 patients may be associated not only with treatment-related factors, such as antibiotic exposure and proton pump inhibitor use, but also with systemic inflammatory response and overall clinical severity. The consistency of these predictors with known clinical mechanisms supports the plausibility of the developed models and highlights the value of combining laboratory, clinical, and treatment-related information for CDI risk stratification.

A key contribution of this study is the integration of predictive modeling with interpretability and explainability methods. SHAP and LIME were used to provide patient-specific explanations of individual model predictions, while odds ratio analysis supported a more global interpretation of feature associations with CDI risk. This combination allowed the system to present not only a predicted probability of CDI, but also the main factors contributing to the prediction. Such explainability is essential in healthcare decision support, where clinicians need to critically evaluate model outputs, understand the reasoning behind predictions, and assess whether the identified risk factors are clinically plausible.

The developed Flask-based web application demonstrates the practical applicability of the proposed approach. By combining CDI risk estimation with transparent, patient-specific explanations, the tool has the potential to support clinicians in identifying hospitalized COVID-19 patients who may require closer monitoring or additional diagnostic attention. Preliminary usability testing indicated good usability and highlighted the value of clear prediction outputs, rapid response, and interpretable explanations. However, these results should be interpreted as an initial proof of concept rather than a completed clinical validation, given the small number of participating physicians and tested clinical cases.

Future work should focus on external multicenter validation, prospective evaluation in real-world clinical workflows, and further refinement of the input feature set. In particular, reducing the number of required variables to those routinely available in hospital information systems would improve practical usability and support broader implementation. Integration with electronic health records could further reduce manual data entry, improve workflow compatibility, and enable more efficient deployment in clinical environments. Overall, the findings suggest that interpretable and explainable machine learning models may support CDI risk stratification and contribute to more transparent, data-driven decision support in hospital care.

## Figures and Tables

**Table 1:** Patient records per pandemic wave.

Wave	Period	Total Patients	CDI Positive	CDI Prevalence
Wuhan	March 2020–February 2021	1124	26	2.31%
Alpha	March 2021–August 2021	824	29	3.52%
Delta	September 2021–December 2021	649	15	2.31%
Omicron	January 2022–May 2024	1251	33	2.64%
Total	—	3848	103	2.68%

**Table 2:** Comparative model performance on the test set.

Model	Dataset	Oversampling	PR-AUC	AUROC (95% CI)	Precision	Recall	F1
RF	MICE	None	0.153	0.798 (0.683–0.913)	0.091	0.667	0.160
XGBoost	MICE	ADASYN	0.141	0.823 (0.713–0.933)	0.089	0.714	0.158
LogReg	KNN	None	0.144	0.776 (0.657–0.895)	0.078	0.619	0.139
MLP	MICE	ROS	0.160	0.706	0.070	0.619	0.125

## Data Availability

The anonymized clinical dataset used in this study is not publicly available due to patient privacy restrictions, institutional data protection policies, and ongoing research activities. Access to the data may be considered by the corresponding author upon reasonable request and subject to approval by the relevant institutional and ethical authorities.

## Supplementary Materials

The following supporting information can be downloaded at https://www.mdpi.com/article/10.3390/healthcare14131869/s1. Table S1: The hyperparameter search spaces used for the evaluated models; Table S2: CDI occurrence across pandemic waves, using the Wuhan wave as the reference category.

## Abbreviations

The following abbreviations are used in this manuscript:

ADASYN	Adaptive Synthetic Sampling
AI	Artificial Intelligence
AUROC	Area Under the Receiver Operating Characteristic Curve
CDI	*Clostridioides difficile* Infection
COVID-19	Coronavirus Disease 2019
CRISP-DM	Cross-Industry Standard Process for Data Mining
CRP	C-Reactive Protein
FDR	False Discovery Rate
ICD-10	International Classification of Diseases, 10th Revision
IL-6	Interleukin-6
KNN	k-Nearest Neighbor
LIME	Local Interpretable Model-Agnostic Explanations
ML	Machine Learning
MLP	Multilayer Perceptron
MICE	Multiple Imputation by Chained Equations
NLR	Neutrophil-to-Lymphocyte Ratio
PR-AUC	Precision–Recall Area Under the Curve
RF	Random Forest
ROC	Receiver Operating Characteristic
ROS	Random OverSampler
SHAP	SHapley Additive exPlanations
SMOTE	Synthetic Minority Oversampling Technique
SUS	System Usability Scale
WBC	White Blood Cell Count
XGBoost	Extreme Gradient Boosting
