# Supplementary material for: Machine Learning Prediction of Clostridioides difficile Infection in Hospitalized COVID-19 Patients Across Pandemic Waves
Source: Healthcare (Basel). 2026 Jun 26;14(13):1869. doi: 10.3390/healthcare14131869 (PMC13362403; doi:10.3390/healthcare14131869)
Supplement: Supplementary file 1 [file healthcare-14-01869-s001.zip › healthcare-4381912-supplementary.pdf]

Table S1. The hyperparameter search spaces used for the evaluated models

Model	Hyperparameter search space
**Random Forest**	n_estimators: 900, 1200, 1600;<br>max_depth: 4, 6, 10, 16;<br>min_samples_split: 10, 20, 40;<br>min_samples_leaf: 4, 8, 12, 20;<br>max_features: sqrt, log2, 0.3, 0.5;<br>class_weight: balanced_subsample, balanced, None;<br>ccp_alpha: 0.0, 0.0001, 0.0003, 0.001, 0.003;<br>min_impurity_decrease: 0.0, 0.0001, 0.0003, 0.001
**XGBoost**	n_estimators: 300, 600, 900, 1200;<br>max_depth: 2, 3, 4, 5, 6;<br>learning_rate: 0.005, 0.01, 0.03, 0.05, 0.1;<br>subsample: 0.6, 0.8, 0.9, 1.0;<br>colsample_bytree: 0.6, 0.8, 0.9, 1.0;<br>reg_lambda: 0.5, 1.0, 2.0, 5.0, 10.0;<br>reg_alpha: 0.0, 0.001, 0.01, 0.1;<br>min_child_weight: 1, 3, 5, 8, 12;<br>gamma: 0, 0.1, 0.3, 0.6, 1.0;<br>max_leaves: 0, 16, 32, 64
**Logistic Regression**	C: 0.003, 0.01, 0.03, 0.1, 0.3, 1.0, 3.0, 10.0, 30.0, 100.0;<br>penalty: l1, l2;<br>tol: 0.0001, 0.0003, 0.001
**Multilayer Perceptron**	hidden_layer_sizes: 32, 64, 64–32, 128–64;<br>alpha: 0.00001, 0.0001, 0.001;<br>learning_rate_init: 0.0001, 0.0003, 0.001

Note: Hyperparameter optimization was performed using RandomizedSearchCV with 5-fold stratified cross-validation and 30 randomly sampled combinations per model. The optimization objective was PR-AUC. Oversampling strategies evaluated within the cross-validation pipeline included no oversampling, RandomOverSampler, SMOTE, and ADASYN.

Table S2. CDI occurrence across pandemic waves, using the Wuhan wave as the reference category

Pandemic wave	Total patients	CDI-positive, n	CDI prevalence, %	Crude OR	95% CI
**Wuhan**	1124	26	2.31	Reference	—
**Alpha**	824	29	3.52	1.54	0.90–2.64
**Delta**	649	15	2.31	1.00	0.53–1.90
**Omicron**	1251	33	2.64	1.14	0.68–1.93

Note: Crude odds ratios were calculated using the Wuhan wave as the reference category. These estimates are unadjusted and should be interpreted as exploratory descriptive associations rather than independent effects of pandemic wave.
